# UPLC-MS/MS-Based Profiling of Eicosanoids in RAW264.7 Cells Treated with Lipopolysaccharide

**DOI:** 10.3390/ijms17040508

**Published:** 2016-04-06

**Authors:** Jae Won Lee, Hyuck Jun Mok, Dae-Young Lee, Seung Cheol Park, Myeong Soon Ban, Jehun Choi, Chun Geon Park, Young-Sup Ahn, Kwang Pyo Kim, Hyung Don Kim

**Affiliations:** 1 Department of Herbal Crop Research, National Institute of Horticultural and Herbal Science, RDA, Eumseong 369-873, Korea; jaewon3@gmail.com (J.W.L.); dylee0809@korea.kr (D.-Y.L.); bms928@nate.com (M.S.B.); jehun@korea.kr (J.C.); pcg@korea.kr (C.G.P.); ay21cay@korea.kr (Y.-S.A.); 2 Department of Applied Chemistry, The Institute of Natural Science, College of Applied Science, Kyung Hee University, Yongin 446-701, Korea; hjmok@khu.ac.kr (H.J.M.); scpark1126@khu.ac.kr (S.C.P.); 3 Department of Biochemistry, School of Life Sciences, Chungbuk National University, Cheongju 361-763, Korea

**Keywords:** eicosanoids, inflammation, macrophage, lipopolysaccharide, UPLC-MS/MS

## Abstract

While both the pro- and anti-inflammatory effects of several eicosanoids have been widely studied, the degree of inflammation in cells that results from various eicosanoids has yet to be comprehensively studied. The objective of this study was to assess the effect of lipopolysaccharide (LPS) treatment on eicosanoid content in RAW264.7 cells. An Ultra performance liquid chromatography coupled to tandem mass spectrometry (UPLC-MS/MS)-based profiling method was used to analyze the eicosanoid contents of RAW264.7 cells treated with different LPS concentrations. The profiling data were subjected to statistical analyses, such as principal component analysis (PCA) and hierarchical clustering analysis. LPS treatment increased nitric oxide production and secretion of pro-inflammatory cytokines, such as tumor necrosis factor-α and interleukin-6, in a concentration-dependent manner. In total, 79 eicosanoids were identified in the cells. RAW264.7 cells treated with different LPS concentrations were well differentiated in the PCA score plot. A heatmap was used to identify the eicosanoids that were up- or down-regulated according to the degree of inflammation and LPS concentration. Thirty-nine eicosanoids were upregulated and seven were down-regulated by LPS treatment in a concentration-dependent manner. Our novel UPLC-MS/MS technique can profile eicosanoids, and can evaluate the correlations between inflammation and eicosanoid metabolism.

## 1. Introduction

**Figure 1:**
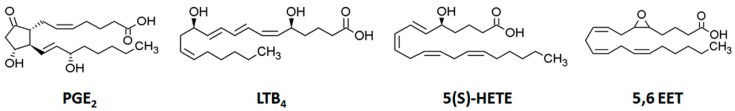
Chemical structures of typical eicosanoids: prostaglandin E_2_ (PGE_2_), leukotriene B_4_ (LTB_4_), 5-hydroxy-eicosatetraenoic acid (5(S)-HETE), and 5,6-epoxy-eicosatrienoic acid (5,6 EET).

Eicosanoids, such as prostaglandins (PGs), leukotrienes (LTs), and a number of hydroxyl and epoxy compounds (Figure 1), are bioactive lipid mediators that play vital roles in physiological and pathophysiological conditions [1,2]. They are key mediators and regulators of inflammation and exert both pro- and anti-inflammatory effects [3,4,5]. Inflammation, a mechanism to protect the host from harmful stimuli, is implicated in the pathogenesis of a number of diseases, including cardiovascular disease, diabetes, allergic diseases, obesity, and cancer [6,7,8,9,10]. Thus, many studies have attempted to identify biomarkers of inflammation, for which the eicosanoids have been targeted as critical metabolites [11,12]. The levels of eicosanoids in biological systems have been assessed to investigate their roles in cell function and pathophysiological events [13,14].

Enzyme-linked immunosorbent assays (ELISAs) are widely used to quantify eicosanoids [15,16]. However, these assays are limited by the lack of commercially available antibodies and their non-specificity. They are also insufficient for multiplex analyses because a large quantity of sample is required to quantify multiple target compounds. For the simultaneous analysis of diverse eicosanoids in a mixture, chromatographic separation using gas chromatography (GC) or liquid chromatography (LC) has been used [17,18]. However, LC is more applicable to analyze various eicosanoids than GC, which requires derivatization to increase the volatility and thermal stability of compounds. Furthermore, ultraviolet (UV) detection lacks sensitivity and is applicable only to a limited number of UV-light-absorbing analytes [19]. Thus, LC coupled to mass spectrometry (MS)-based techniques have been recently used to profile eicosanoids [20,21,22,23]. Ultra-performance LC (UPLC) with a small-particle-size column provides fast and effective separation of various molecules [24]. Multiple reaction monitoring (MRM) based on tandem MS (MS/MS) is also effective for the sensitive and selective profiling of each molecular species [25]. Finally, UPLC-MS/MS is a good tool for the comprehensive profiling of eicosanoids. Such characterization of eicosanoids may provide insight into their correlations with inflammatory conditions.

Macrophages, which are the major source of various lipid mediators, respond to a variety of stimuli by producing various eicosanoids [26,27]. The RAW264.7 murine macrophage cell line has been used in numerous studies as a model of primary macrophages. Lipopolysaccharide (LPS) induces cytokine production and the acute-phase response, and so has been used extensively in mechanistic studies of inflammation [28,29]. In this study, UPLC-MS/MS was used to profile the eicosanoid content of RAW264.7 cells treated with various concentrations of LPS. The degree of cellular inflammation may differ depending on LPS concentration. The objective of this study was to perform a comprehensive profiling of eicosanoids known to be related to inflammation as well as other less known species in inflammatory cells. The characterization of eicosanoids altered in RAW264.7 cells by different levels of inflammation may help elucidate the correlation between inflammation and eicosanoid metabolism.

## 2. Results

### 2.1. Effect of Lipopolysaccharide (LPS) on RAW264.7 Cells

**Figure 2:**
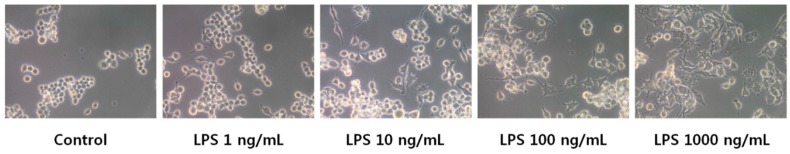
Morphological changes in RAW264.7 cells treated with 0, 1, 10, 100, and 1000 ng/mL lipopolysaccharide (LPS). The morphology of RAW 264.7 cells was visualized by optical microscopy (400×).

**Figure 3:**
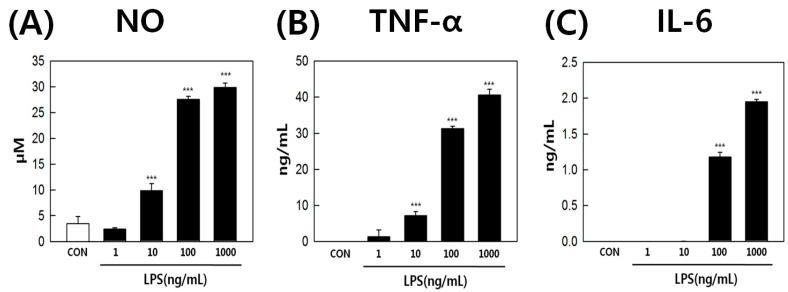
(**A**) nitric oxide (NO); (**B**) tumor necrosis factor-α (TNF-α); and (**C**) interleukin-6 (IL-6) production by RAW264.7 cells treated with 0, 1, 10, 100, and 1000 ng/mL LPS. *** *p* value < 0.001.

LPS-stimulated RAW264.7 cells were used to elucidate the correlation between inflammation and eicosanoid metabolism. The cells were treated with 0, 1, 10, 100, 1000 ng/mL LPS for 18 h, and monitored under optical microscopy. LPS is known to induce the differentiation of RAW264.7 cells into dendritic-like cells [30,31]. We observed that LPS-activated RAW264.7 cells had a differentiated form with accelerated spreading and forming pseudopodia (Figure 2). Next, the supernatants were subjected to quantification of nitric oxide (NO) and pro-inflammatory cytokine levels. LPS-induced NO production in RAW264.7 cells changed in a concentration-dependent manner (Figure 3A). NO production by cells treated with 1000 ng/mL LPS was 10-fold greater than that of the control. LPS also induced the secretion of pro-inflammatory cytokines, such as tumor necrosis factor-α (TNF-α) and interleukin-6 (IL-6), by RAW264.7 cells in a concentration-dependent fashion (Figure 3B,C). These results showed that LPS activated inflammatory signaling pathways.

### 2.2. Profiling of Eicosanoids by Ultra Performance Liquid Chromatography Coupled to Tandem Mass Spectrometry (UPLC-MS/MS)

**Figure 4:**
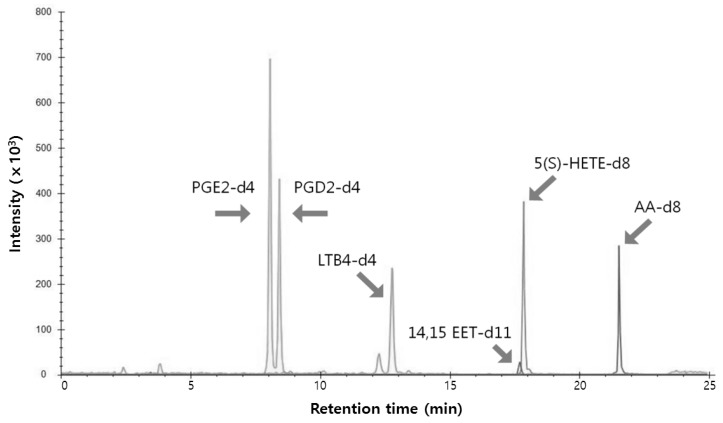
Merged multiple reaction monitoring data of six standards—PGE_2_-d4, PGD_2_-d4, LTB_4_-d4, 14,15-EET-d11, 5(S)-HETE-d8, and AA-d8.

To profile the eicosanoid levels in RAW264.7 cells, we established a multiple-reaction monitoring (MRM) method based on UPLC-MS/MS. First, several standards, 5-hydroxy-eicosatetraenoic acid-d8 (5(S)HETE-d8), 14,15-epoxy-eicosatrienoic acid-d11 (14,15EET-d11), leukotriene B_4_-d4 (LTB_4_-d4), prostaglandin E_2_-d4 (PGE_2_-d4), prostaglandin D_2_-d4 (PGD_2_-d4), and arachidonic acid-d8 (AA-d8), were used to optimize the analytical conditions. The adduct ion of each eicosanoid was confirmed by MS scans and were detected primarily as [M ‒ H]^−^ ions by electropray ionization (ESI) in the negative-ion mode. Previously reported MRM transitions for eicosanoids were employed in the current study [32]. Furthermore, source conditions (source temperature (Temp), nebulizer gas (GS1), heater gas (GS2)) and the compound parameters (collision energy (CE) and declustering potential (DP)) of the mass spectrometer were optimized using eicosanoid standards. Table 1 shows the MRM transitions (precursor *m*/*z* (Q1) > product *m*/*z* (Q3)), DP, and CE of the eicosanoid standards. Use of a UPLC system with a small-particle-size column enabled effective separation of these standards within 25 min at a flow rate of 0.5 mL/min (Figure 4).

Next, the performance of eicosanoid analyses based on MRM was validated (Table 2). Each eicosanoid standard was analyzed six times and the relative standard deviations (RSDs) (%) were calculated. The RSDs (%) of the relative RTs and the relative peak areas were less than 0.3% and 8%, respectively. This method showed a high degree of reproducibility and the correlation coefficient (*R*^2^) for each eicosanoid analysis was at least 0.9959, indicating a high degree of reliability. The limit of detection (LOD) and the limit of quantification (LOQ) of the eicosanoid standards are also shown. With the exception of 14,15-EET-d11, the LC/MS method exhibited sufficient sensitivity for the standards. Thus, in the analysis of biological samples, a higher concentration of 14,15-EET-d11 was required for its use as an internal standard (IS).

### 2.3. Quantification of Eicosanoids in LPS-Treated RAW264.7 Cells

The above UPLC/MS/MS-based method was used to quantify eicosanoids in RAW264.7 cells treated with 0, 1, 10, 100, and 1000 ng/mL LPS. Assays were performed in triplicate for each LPS concentration. Eicosanoids were extracted using 10% methanol. In this study, a total of 150 MRM transitions were used to profile eicosanoids. A total of 79 eicosanoids were identified in RAW264.7 cells (Table 3). To ensure reliable quantification, we used deuterated compounds, including 5(S)-HETE-d8, 14,15-EET-d11, AA-d8, LTB_4_-d4, and PGE_2_-d4, as ISs to normalize the data. The normalized data (peak areas of compounds/peak area of IS) were subjected to statistical analyses.

**Figure 5:**
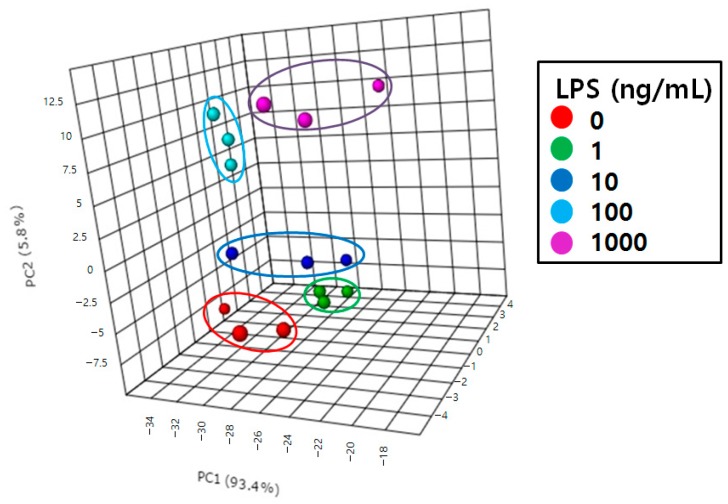
Principal component analysis (PCA) score plot of RAW264.7 cells treated with 0, 1, 10, 100, and 1000 ng/mL LPS.

First, we applied principal component analysis (PCA) [33,34] to differentiate the RAW264.7 cells treated with various LPS concentrations. This resulted in effective separation in the corresponding PCA score plot (Figure 5). Each point represents an individual sample and the scatter of samples indicates similarities or differences in eicosanoid composition. Samples treated with 0, 1, and 10 ng/mL LPS were scattered on the lower side of the plot and those treated with 100 and 1000 ng/mL LPS were scattered on the upper side. The degree of inflammation increased with increasing LPS concentration. This demonstrated that eicosanoid contents differ depending on the degree of inflammation.

**Figure 6:**
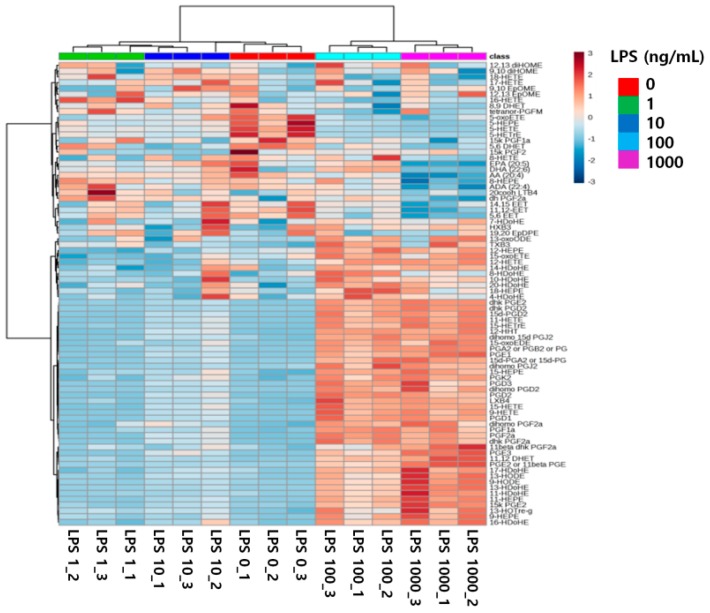
Hierarchical clustering of 79 eicosanoid datasets from RAW264.7 cells treated with 0, 1, 10, 100, and 1000 ng/mL LPS.

Second, changes in the levels of 79 eicosanoids can be described in a heatmap (Figure 6). This hierarchical clustering enabled effective differentiation of the five groups. Several of the 79 eicosanoid species were upregulated by treatments with 100 and 1000 ng/mL LPS. In contrast, the levels of other eicosanoids decreased with increasing LPS concentration. The levels of still other species were not correlated with LPS concentration. Therefore, up- and down-regulation of eicosanoids were associated with LPS-induced inflammation.

## 3. Discussion

**Figure 7:**
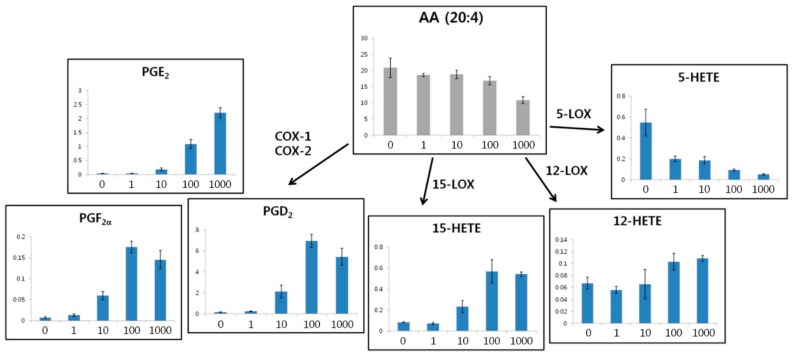
The eicosanoid synthesis pathway from arachidonic acid (AA) and eicosanoid levels in RAW264.7 cells as a function of LPS concentration (0, 1, 10, 100, and 1000 ng/mL). COX, cyclooxygenase; LOX, lipoxygenase; PG, prostaglandin; HETE, hydroxyeicosatetraenoic acid.

To evaluate altered eicosanoid metabolism in RAW264.7 cells, we focused on the pathway of eicosanoid synthesis from AA (Figure 7). Several PGs, such as PGD_2_, PGE_2_, and PGF_2α_, are synthesized from AA by cyclooxygenase (COX)-1 and COX-2. Furthermore, 15-HETE is synthesized from AA by 15-lipoxygenase (LOX). In addition, 12-LOX and 5-LOX also synthesize 12-HETE and 5-HETE, respectively, from AA. Our results showed that AA was down-regulated following treatment with high concentrations of LPS. This indicated that AA was used as the substrate to synthesize several eicosanoids. PGE_2_, which plays a pro-inflammatory role [35,36], was upregulated following treatment with high concentrations of LPS. In contrast, two other PGs (PGE_2_ and PGF_2α_) and 15-HETE were upregulated. In addition, 12-HETE was upregulated slightly and 5-HETE was down-regulated following treatments with high concentrations of LPS. Therefore, COX-1, COX-2, and 15-LOX were highly activated by LPS-induced inflammation, whereas 12-LOX was only slightly activated and 5-LOX was suppressed. 

Many previous studies have reported the utility of various eicosanoid species as biomarkers for diseases and pathophysiological conditions [11,37,38,39]. For example, the endogenous levels of AA, PGE_2_, and 12-HETE were significantly altered in cancerous mucosae [40]. This indicates that inflammation is correlated with tumorigenesis. Rago *et al.* [41] also reported the quantities of several eicosanoids in human plasma to develop biomarkers to distinguish three groups: healthy individuals, hypertensive patients, and severe atherosclerotic patients. The results showed that lower levels of 8-HETE, LTB4, 9-HODE, and 13-HODE are potential biomarkers for severe heart disease. Eicosanoid metabolism may differ depending on biological samples and pathophysiological events. However, the detailed metabolism of major and minor eicosanoids in human diseases that involve inflammation has yet to be studied.

To characterize eicosanoid metabolism in inflammatory cells, comprehensive profiling of various eicosanoids is required. In this study, we evaluated changes in the levels of not only known eicosanoids related to inflammation but also other, less well-known species. The following 39 eicosanoids were upregulated following treatment with high concentrations of LPS: (1) eicosanoids derived from AA: 9-HETE, 11-HETE, 12-HETE, 15-HETE, 11,12-DHET, 12-HHT, PGA_2_, PGD_2_, PGE_2_, PGF_2α_, dihomo PGD_2_, dihomo PGF_2α_, dihomo PGJ_2_, dihomo 15d PGJ_2_, 15d-PGA_2_, 15d-PGD_2_, 11β dhk PGF_2α_, dhk PGD_2_, dhk PGE_2_, PGK_2_, 15k PGF_2_, and LXB_4_; (2) eicosanoids derived from linoleic acid: 9-HODE, and 13-HODE; (3) eicosanoids derived from eicosapentaenoic acid: 9-HEPE, 11-HEPE, 15-HEPE, 15-oxoEDE, PGD_3_, and PGE_3_; (4) eicosanoids derived from docohexaenoic acid: 11-HDoHE, 13-HDoHE, 16-HDoHE, and 17-HDoHE; (5) eicosanoids derived from gamma-linoleic acid: 13-HOTre-g; and (6) eicosanoids derived from dihomo-gamma-linoleic acid: 15-HETrE, PGD_1_, PGE_1_, and PGF_1α_. The following seven eicosanoids were down-regulated following treatments with high concentrations of LPS: (1) eicosanoids derived from AA: 5-HETE, 5,6-DHET, 5,6-EET, and 11,12-EET; (2) eicosanoids derived from eicosapentaenoic acid: 5-HEPE; and (3) eicosanoids derived from dihomo-gamma-linoleic acid: 5-HETrE. Other eicosanoid species were not influenced by LPA treatment in a concentration-dependent manner.

In conclusion, a profiling method based on UPLC-MS/MS was used to characterize the effect of LPS treatment on eicosanoid metabolism in RAW264.7 cells. The degree of inflammation increased with increasing LPS concentration. A total of 79 eicosanoids were identified in RAW264.7 cells. PCA and heatmap generation were used to differentiate RAW264.7 cells treated with different concentrations of LPS. The five groups were well separated in the PCA score plot, and the heatmap was used to identify the up- or downregulation of eicosanoids according to LPS concentration. To our knowledge, this study is the first attempt to assess the levels of cellular eicosanoids altered by the degree of inflammation. A total of 39 eicosanoids were upregulated, and seven were down-regulated by LPS treatment in a concentration-dependent manner. Our novel UPLC-MS/MS technique has the potential for eicosanoid profiling and evaluating correlations between inflammation and eicosanoid metabolism. The eicosanoids up- or down-regulated by LPS can be applied as typical biomarkers for inflammation. In the future, the levels of inflammation-related eicosanoids in biological samples are needed to estimate their roles in cell function and pathophysiological events.

## 4. Materials and Methods

### 4.1. Reagents

HPLC-grade water, methanol, acetonitrile, and isopropyl alcohol were purchased from J.T. Baker (Avantor Performance Material, Inc., Center Valley, PA, USA). Acetic acid and ammonium acetate were obtained from Sigma-Aldrich (St. Louis, MO, USA). The eicosanoid standards were as follows: 14,15-EET-d11, 5(S)HETE-d8, LTB_4_-d4, PGE_2_-d4, PGD_2_-d4, and AA-d8 (Cayman Chemical, Ann Arbor, MI, USA). A Strata-x 33-µm polymerized solid reverse-phase extraction column (cat # 8B-S100-UBJ) was purchased from Phenomenex (Torrance, CA, USA). Dulbecco’s modified Eagle’s medium (DMEM), fetal bovine serum (FBS), penicillin-streptomycin, and trypsin-ethylenediaminetetraacetic acid (EDTA) were purchased from HyClone Laboratories Inc. (Logan, UT, USA). Escherichia coli LPS and Griess reagent were obtained from Sigma Chemical Co. (St Louis, MO, USA).

### 4.2. Cell Culture and LPS Treatment

Murine macrophage RAW264.7 cells (KCLB 40071; Korean Cell Line Bank, Seoul, Korea) were cultured in DMEM containing 10% heat-inactivated FBS, 100 U/mL penicillin, and 100 µg/mL streptomycin in a humidified atmosphere with 5% CO_2_ at 37 °C. Cells were seeded in a 100-mm Petri dish, cultured for 24 h, and incubated for a further 18 h after treatment with 100 µL LPS (0, 1, 10, 100, and 1000 ng/mL). Cell pellets were collected from each culture dish and the cells were enumerated using a hemocytometer. Finally, 1 × 10^7^ cells were used in the analyses.

### 4.3. Nitric Oxide (NO) and Enzyme-Linked Immunosorbent Assays (ELISAs) Analyses

NO concentrations in the culture supernatants were determined using a spectrophotometric assay based on the Griess reaction. A calibration curve was constructed using known concentrations (0–100 µM) of sodium nitrite. Levels of proinflammatory cytokines such as TNF-α and IL-6 were analyzed using commercial ELISA kits (BD Biosciences, San Diego, CA, USA) according to the manufacturer’s instructions.

### 4.4. Sample Preparation

A simple solid-phase extraction method was applied for the extraction of eicosanoids [25]. Cells were resuspended in 1 mL of 10% methanol in water (*v*/*v*) and sonicated for 5 min. Samples were then spiked with 10 ng of deuterated internal standards. Eicosanoids were extracted using Strata-x 33-µm polymerized solid reverse-phase extraction columns. Briefly, the columns were activated with 3.5 mL of 100% methanol followed by 3.5 mL of water for equilibration. After loading the samples, the columns were washed with 3.5 mL of 10% methanol in water to remove non-specific-binding metabolites. Eicosanoids were eluted into 1 mL methanol. The eluted samples were dried using a Speed-Vac concentrator (Labconco, Kansas City, MO, USA) and resuspended in 90 µL of Solvent A, as described below. The samples were stored at −80 °C until analysis.

### 4.5. UPLC-MS/MS Conditions

The UPLC analyses were performed on a Waters ACQUITY UPLC instrument (Waters Corp., Milford, MA, USA). The temperature of the column oven and autosampler were set at 40 and 4 °C, respectively. An Acquity BEH300 C18 column (2.1 × 150 mm ID; 1.7 µm; Waters Corp.) was used for the separation of eicosanoids. Solvent A consisted of water/acetonitrile/acetic acid (70:30:0.02; *v*/*v*/*v*) and solvent B of acetonitrile/isopropyl alcohol (50:50, *v*/*v*). The gradient elution program was as follows: 0–1 min, B 0%; 1–3 min, B 0%–25%; 3–11 min, B 25%–45%; 11–13 min, B 45%–60%; 13–18 min, B 60%–75%; 18–18.5 min, B 75%–90%; 18.5–20 min, B 90%; and 20–21 min, B 90%–0%. The column was equilibrated with 0% Solvent B for 4 min prior to analysis of the next sample. The total run time was 25 min for each analysis. The flow rate was 0.5 mL/min and the injection volume was 40 µL for each run.

For the MS analyses, an ABI/Sciex (Foster City, CA, USA) 5500 QTRAP hybrid, triple quadrupole, linear ion trap mass spectrometer equipped with a Turbo V ion source, together with the Analyst 1.5.1 software package (ABI/Sciex, Foster City, CA, USA), was used. Ultra-pure nitrogen gas was used as the collision gas for eicosanoids. The typical operating source conditions for the analysis of eicosanoids in negative ion ESI mode were optimized using deuterated standards as follows: curtain gas (CUR) = 10 psi, GS1 = 30 psi, GS2 = 30 psi, ionspray voltage (IS) = −4500 V, collision gas setting (CAD) = high, Temp = 525 °C, Ihe = on, entrance potential (EP) = −10 V, and collision cell exit potential (CXP) = −10 V.

### 4.6. Validation Study

External standard curves were established from the analysis of mixed eicosanoid standards at 16 different concentrations (0.1, 0.3, 0.6, 1, 3, 6, 10, 30, 60, 100, 300, 600, 1000, 3000, 6000, and 10,000 pg). In the case of 14,15 EET-d11, seven different concentrations (1000, 3000, 6000, 10,000, 30,000, 60,000, and 100,000 pg) were applied. The LOD, LOQ, linear ranges, and correlation (*R*^2^) values were determined by the external standard curve. The RSDs (%) of the relative RTs and the relative peak areas were estimated after six replicate analyses of 3000 pg of each standard.

### 4.7. Data Processing and Statistical Analysis

LC-MS data were obtained using the Analyst 1.5.1 software package (ABI/Sciex, Foster City, CA, USA). Eicosanoid peaks were assigned by comparisons of retention times with those of the internal standards. The Skyline software package (MacCoss Laboratory, University of Washington, Seattle, WA, USA) was used as an in-house database to determine the peak area of each assigned lipid from replicate raw data. The extracted peak areas of lipid peaks were normalized to the appropriate internal standard [42]. Hierarchical clustering of the quantified eicosanoids and PCA analyses was performed on the MetaboAnalyst web site [43].

## Figures and Tables

**Table 1:** Optimized multiple-reaction monitoring (MRM) conditions for eicosanoid standards.

Compounds	Abbreviation	Ion Mode	MRM Transitions		DP	CE
			Q1	Q3		
Prostaglandin E_2_-d4	PGE_2_-d4	Negative	355	275	−50	−25
Prostaglandin D_2_-d4	PGD_2_-d4	Negative	355	275	−50	−25
Leukotriene B_4_-d4	LTB_4_-d4	Negative	339	197	−70	−22
14,15 Epoxy-eicosatrienoic acid-d11	14,15 EET-d11	Negative	330	202	−50	−15
5-Hydroxy-eicosatetraenoic acid-d8	5(S)-HETE-d8	Negative	327	116	−50	−20
Arachidonic acid-d8	AA-d8	Negative	311	267	−80	−20

Q1, precursor *m*/*z*; Q3, product *m*/*z*; DP, Declustering potential; CE, Collision energy.

**Table 2:** Validation of the eicosanoid profiling method based on UPLC-MS/MS and the LOD and LOQ of each compound.

Eicosanoids	RT (min)	RSD (*n* = 6) (%)		Correlation (*R*^2^)	Linear Range (pg)	LOD (pg)	LOQ (pg)
		RT	Peak Area				
PGE_2_-d4	8.06	0.25	2.85	0.9978	30–10,000	3	30
PGD_2_-d4	8.43	0	4.44	0.9982	30–10,000	3	30
LTB_4_-d4	12.77	0.20	5.39	0.9969	100–10,000	60	100
14,15 EET-d11	17.70	0	6.08	0.9964	10,000–100,000	3000	10,000
5(S)-HETE-d8	17.85	0	5.01	0.9972	30–10,000	6	30
AA-d8	21.53	0	7.84	0.9959	3–6000	0.1	3

UPLC-MS/MS, Ultra performance liquid chromatography coupled to tandem mass spectrometry; LOD, The limit of detection; LOQ, The limit of quantification; RT, Retention time; RSD, Relative standard deviation.

**Table 3:** Eicosanoids identified in RAW264.7 cells.

No.	Compound Name	Abbreviation	MRM Transitions		DP	CE	RT (min)	IS	Alteration by LPS Treatment
			Q1	Q3					
1	12S-hydroxy-heptadecatrienoic acid	12-HHT	279	163	−30	−30	14.3	5(S)HETE-d8	Up
2	13-hydroxy-g-octadecatrienoic acid	13-HOTre-g	293	193	−70	−20	15.5	5(S)HETE-d8	Up
3	9-hydroxy-octadecadienoic acid	9-HODE	295	171	−60	−25	16.6	5(S)HETE-d8	Up
4	13-hydroxy-octadecadienoic acid	13-HODE	295	195	−60	−25	16.3	5(S)HETE-d8	–
5	9,10-hydroxy-octadecadienoic acid	9,10-diHOME	313	201	−60	−30	14.2	5(S)HETE-d8	–
6	12,13-hydroxy-octadecadienoic acid	12,13-diHOME	313	183	−60	−30	13.5	5(S)HETE-d8	–
7	9-hydroxy-eicosapentaenoic acid	9-HEPE	317	149	−75	−20	15.7	5(S)HETE-d8	Up
8	5-hydroxy-eicosapentaenoic acid	5-HEPE	317	115	−40	−17	16.6	5(S)HETE-d8	Down
9	15-hydroxy-eicosapentaenoic acid	15-HEPE	317	219	−60	−20	15.5	5(S)HETE-d8	Up
10	8-hydroxy-eicosapentaenoic acid	8-HEPE	317	127	−70	−25	15.5	5(S)HETE-d8	–
11	11-hydroxy-eicosapentaenoic acid	11-HEPE	317	121	−70	−24	15.7	5(S)HETE-d8	Up
12	12-hydroxy-eicosapentaenoic acid	12-HEPE	317	179	−70	−20	15.9	5(S)HETE-d8	–
13	18-hydroxy-eicosapentaenoic acid	18-HEPE	317	215	−60	−20	15.1	5(S)HETE-d8	–
14	11-hydroxy-eicosatetraenoic acid	11-HETE	319	167	−60	−20	17.1	5(S)HETE-d8	Up
15	9-hydroxy-eicosatetraenoic acid	9-HETE	319	151	−60	−20	17.0	5(S)HETE-d8	Up
16	5-hydroxy-eicosatetraenoic acid	5-HETE	319	115	−60	−20	17.9	5(S)HETE-d8	Down
17	8-hydroxy-eicosatetraenoic acid	8-HETE	319	155	−60	−20	17.3	5(S)HETE-d8	–
18	15-hydroxy-eicosatetraenoic acid	15-HETE	319	219	−50	−15	16.7	5(S)HETE-d8	Up
19	12-hydroxy-eicosatetraenoic acid	12-HETE	319	179	−60	−20	17.3	5(S)HETE-d8	Up
20	18-hydroxy-eicosatetraenoic acid	18-HETE	319	261	−80	−25	15.9	5(S)HETE-d8	–
21	17-hydroxy-eicosatetraenoic acid	17-HETE	319	247	−80	−25	15.9	5(S)HETE-d8	–
22	16-hydroxy-eicosatetraenoic acid	16-HETE	319	189	−80	−25	16.0	5(S)HETE-d8	–
23	5-hydroxy-eicosatrienoic acid	5-HETrE	321	115	−70	−19	19.1	5(S)HETE-d8	Down
24	15-hydroxy-eicosatrienoic acid	15-HETrE	321	221	−70	−21	17.4	5(S)HETE-d8	Up
25	5,6-dihydroxy-eicosatrienoic acid	5,6-DHET	337	145	−75	−25	16.5	5(S)HETE-d8	Down
26	8,9-dihydroxy-eicosatrienoic acid	8,9-DHET	337	127	−60	−30	15.5	5(S)HETE-d8	–
27	11,12-dihydroxy-eicosatrienoic acid	11,12-DHET	337	167	−60	−25	15.8	5(S)HETE-d8	Up
28	8-hydroxy-docosahexaenoic acid	8-HDoHE	343	109	−70	−20	17.5	5(S)HETE-d8	–
29	7-hydroxy-docosahexaenoic acid	7-HDoHE	343	141	−60	−18	17.3	5(S)HETE-d8	–
30	4-hydroxy-docosahexaenoic acid	4-HDoHE	343	101	−70	−17	18.2	5(S)HETE-d8	–
31	10-hydroxy-docosahexaenoic acid	10-HDoHE	343	181	−60	−17	16.9	5(S)HETE-d8	–
32	11-hydroxy-docosahexaenoic acid	11-HDoHE	343	149	−60	−19	17.0	5(S)HETE-d8	Up
33	13-hydroxy-docosahexaenoic acid	13-HDoHE	343	221	−60	−17	16.7	5(S)HETE-d8	Up
34	16-hydroxy-docosahexaenoic acid	16-HDoHE	343	233	−75	−19	16.5	5(S)HETE-d8	Up
35	20-hydroxy-docosahexaenoic acid	20-HDoHE	343	241	−60	−20	16.3	5(S)HETE-d8	–
36	17-hydroxy-docosahexaenoic acid	17-HDoHE	343	245	−60	−20	16.5	5(S)HETE-d8	Up
37	14-hydroxy-docosahexaenoic acid	14-HDoHE	343	205	−60	−18	16.7	5(S)HETE-d8	–
38	Arachidonic acid	AA	303	259	−80	−20	21.6	AA-d8	Down
39	Eicosapentaenoic acid	EPA	301	257	−65	−15	20.4	AA-d8	–
40	Adrenic acid	ADA	331	287	−80	−20	22.3	AA-d8	–
41	Dohexacosaenoic acid	DHA	327	283	−95	−20	21.3	AA-d8	–
42	13-oxo-octadecadienoic acid	13-oxoODE	293	113	−70	−30	16.6	14,15 EET-d11	–
43	9,10-epoxy-octadecenoic acid	9,10-EpOME	295	171	−60	−25	18.4	14,15 EET-d11	–
44	12,13-epoxy-octadecenoic acid	12,13-EpOME	295	195	−60	−25	18.1	14,15 EET-d11	–
45	5-5-oxo-eicosatetraenoic acid	5-oxoETE	317	203	−40	−25	18.3	14,15 EET-d11	–
46	15-5-oxo-eicosatetraenoic acid	15-oxoETE	317	113	−40	−25	16.7	14,15 EET-d11	–
47	11,12-epoxy-eicosatrienoic acid	11,12-EET	319	167	−60	−20	18.6	14,15 EET-d11	Down
48	14,15-epoxy-eicosatrienoic acid	14,15-EET	319	219	−50	−15	18.1	14,15 EET-d11	–
49	5,6-epoxy-eicosatrienoic acid	5,6-EET	319	191	−30	−20	18.8	14,15 EET-d11	Down
50	15-oxo-eicosadienoic acid	15-oxoEDE	321	113	−100	−32	18.1	14,15 EET-d11	Up
51	Hepoxilin B_3_	HXB_3_	335	183	−40	−20	15.5	14,15 EET-d11	–
52	19,20-epoxy Docosapentaenoic acid	19,20-EpDPE	343	241	−60	−20	17.6	14,15 EET-d11	–
53	Lipoxin B_4_	LXB_4_	351	221	−80	−25	8.4	LTB_4_-d4	Up
54	20-carboxy-Leukotriene B_4_	20cooh LTB_4_	365	195	−60	−25	6.4	LTB_4_-d4	–
55	15-deoxy-Prostaglandin A_2_ or 15-deoxy-Δ12,14-PGJ_2_	15d-PGA_2_ or 15d-PGJ_2_	315	271	−50	−15	15.2	PGE_2_-d4	Up
56	Tetranor-Prostanglin F Metabolite	tetranor-PGFM	329	293	−40	−25	3.1	PGE_2_-d4	–
57	Prostaglandin A_2_ or Prostaglandin B_2_ or Prostaglandin J_2_	PGA_2_ or PGB_2_ or PGJ_2_	333	271	−30	−20	10.5	PGE_2_-d4	Up
58	15-deoxy-Δ12,14-PGD_2_	15d-PGD_2_	333	271	−30	−20	12.8	PGE_2_-d4	Up
59	Prostaglandin D_3_	PGD_3_	349	269	−55	−25	7.4	PGE_2_-d4	Up
60	Prostaglandin E_3_	PGE_3_	349	269	−55	−25	7.1	PGE_2_-d4	Up
61	15-keto-Prostaglandin E_2_	15k PGE_2_	349	113	−35	−30	8.3	PGE_2_-d4	–
62	Prostaglandin K_2_	PGK_2_	349	205	−50	−30	8.3	PGE_2_-d4	Up
63	15-keto-Prostaglandin F_2_	15k PGF_2_	351	113	−40	−35	8.6	PGE_2_-d4	Up
64	Prostaglandin E_2_	PGE_2_	351	271	−50	-25	8.1	PGE_2_-d4	Up
65	Prostaglandin D_2_	PGD_2_	351	271	−50	−25	8.4	PGE_2_-d4	Up
66	13,14-dihydro-15-keto Prostaglandin E_2_	dhk PGE_2_	351	207	−40	−25	8.4	PGE_2_-d4	Up
67	13,14-dihydro-15-keto Prostaglandin D_2_	dhk PGD_2_	351	207	−40	−25	9.3	PGE_2_-d4	Up
68	Prostaglandin F_2α_	PGF_2α_	353	193	−50	−30	8.5	PGE_2_-d4	Up
69	15-keto-Prostaglandin F_1α_	15k PGF_1α_	353	113	−50	−35	3.1	PGE_2_-d4	–
70	11β-13,14-dihydro-15-keto-Prostaglandin F_2α_	11β dhk PGF_2α_	353	113	−50	−35	9.3	PGE_2_-d4	Up
71	Prostaglandin E_1_	PGE_1_	353	273	−55	−25	8.1	PGE_2_-d4	Up
72	Prostaglandin D_1_	PGD_1_	353	273	−55	−25	8.5	PGE_2_-d4	Up
73	Prostaglandin F_1α_	PGF_1α_	355	293	−75	−30	8.2	PGE_2_-d4	Up
74	13,14-dihydro-Prostaglandin F_2α_	dh PGF_2α_	355	275	−40	−25	8.9	PGE_2_-d4	–
75	Dihomo-Prostaglandin J_2_	Dihomo-PGJ_2_	361	299	−55	−25	13.0	PGE_2_-d4	Up
76	Dihomo-15-deoxy-Prostaglandin J_2_	Dihomo-15d PGJ_2_	361	299	−55	−25	14.4	PGE_2_-d4	Up
77	Thromboxane B_3_	TXB_3_	367	169	−50	−25	6.4	PGE_2_-d4	–
78	Dihomo-Prostaglandin F_2α_	Dihomo-PGF_2α_	381	221	−75	−35	10.0	PGE_2_-d4	Up
79	Dihomo-Prostaglandin D_2_	Dihomo-PGD_2_	379	299	−65	−30	10.4	PGE_2_-d4	Up

## Abbreviations

LPS	Lipopolysaccharide
UPLC	Ultra performance liquid chromatography
MS	Mass spectrometry
PG	Prostaglandin
LT	Leukotriene
ELISA	Enzyme-linked immunosorbent assays
NO	Nitric oxide
TNF-α	Tumor necrosis factor-α
IL-6	Interleukin-6
MRM	Multiple reaction monitoring
CE	Collision energy
DP	Declustering potential
RSD	Relative standard deviation
LOD	The limit of detection
LOQ	The limit of quantification
IS	Internal standard
PCA	Principal component analysis
EDTA	Ethylenediaminetetraacetic acid
